# Supplementary material for: Predicting the Pathway Involvement of Compounds Annotated in the Reactome Knowledgebase
Source: Metabolites. 2025 Mar 1;15(3):161. doi: 10.3390/metabo15030161 (PMC11944036; doi:10.3390/metabo15030161)
Supplement: Supplementary file 1 [file metabolites-15-00161-s001.zip › metabolites-3497450-supplementary.pdf]

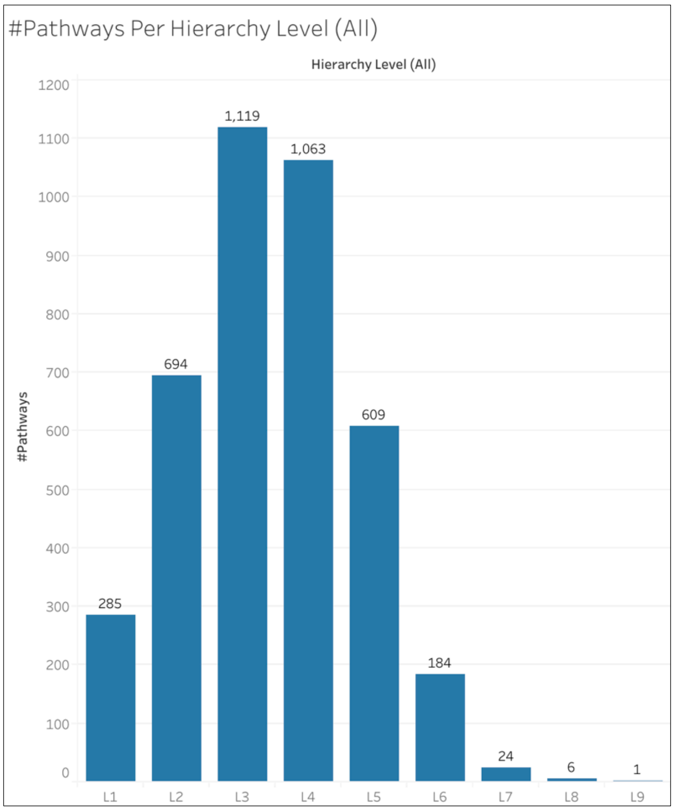

Figure S1. Number of Pathways in Each Pathway Hierarchy Level.

Table S1. Scores for All Metrics by Pathway Hierarchy Levels Included in

Pathway Hierarchy Levels Included	Metric	Mean Score	Median Score	Standard Deviation
L1+	Accuracy	0.997	0.998	0.0006
	F1 Score	0.915	0.918	0.0161
	MCC	0.916	0.919	0.0149
	Precision	0.857	0.862	0.0304
	Recall	0.982	0.982	0.0049
	Specificity	0.998	0.998	0.0006
L2+	Accuracy	0.998	0.998	0.0003
	F1 Score	0.905	0.905	0.0107
	MCC	0.907	0.907	0.0099
	Precision	0.842	0.842	0.0209
	Recall	0.978	0.979	0.0039
	Specificity	0.998	0.998	0.0003
L3+	Accuracy	0.998	0.998	0.0003
	F1 Score	0.881	0.883	0.0148
	MCC	0.884	0.886	0.0134
	Precision	0.805	0.807	0.0267
	Recall	0.974	0.974	0.0047
	Specificity	0.998	0.998	0.0004

Table S2. oMCC and Confusion Matrix Counts by the Pathway Hierarchy Levels Included in the Dataset and the Hierarchy Level in the Test Set.

Hierarchy Levels Included	Hierarchy Level	oMCC	True positives	True negatives	False positives	False negatives
L1+	1	0.938	563,996	10,619,088	65,049	7,234

	2	0.930	582,623	26,759,305	80,134	7,531
	3	0.911	514,134	43,613,980	91,202	10,241
	4	0.892	330,781	41,603,659	73,268	8,829
	5	0.869	141,065	23,876,858	39,419	4,681
	6	0.836	41,892	7,209,849	15,296	1,894
	7	0.835	4,427	941,614	1,524	290
	8	0.833	1,519	235,409	485	134
	9	0.937	126	39,647	14	3
**L2+**	2	0.922	144,874	6,685,868	21,619	2,740
	3	0.911	128,282	10,898,556	22,922	2,440
	4	0.896	83,089	10,402,737	17,739	2,100
	5	0.874	35,067	5,973,762	9,443	1,065
	6	0.841	10,664	1,802,425	3,718	478
	7	0.827	1,122	235,817	403	81
	8	0.857	379	58,768	99	29
	9	0.987	39	9,674	0	1
**L3+**	3	0.894	126,895	10,895,972	27,108	3,556
	4	0.886	83,253	10,400,231	20,170	1,931
	5	0.863	35,620	5,970,538	10,797	990
	6	0.824	10,303	1,800,444	4,274	399
	7	0.823	1,164	236,108	456	68
	8	0.838	406	58,744	131	30
	9	0.960	35	9,674	3	0
